# A survey on providers’ views on utilization of dental hygienists and dental therapists delivering periodontal care in the South West of England

**DOI:** 10.1038/s41405-025-00372-2

**Published:** 2025-12-16

**Authors:** Cher Farrugia, Janita Dhariwal, Chris Bell, Ana Beatriz Oliveira Gambôa

**Affiliations:** Bristol Dental School, University of Bristol, Bristol, UK

**Keywords:** Periodontics, Periodontitis

## Abstract

**Aim:**

To examine the opportunities and limitations for dental hygienists and therapists (DCPs) delivering periodontal care in primary care settings.

**Materials and Methods:**

A cross-sectional survey was conducted among primary care dental providers in the South West of England following University of Bristol ethical approval (number 15281). The survey assessed the current providers’ views on utilization of DCPs, including availability, their practice, direct access and provider perspectives on referrals and remuneration.

**Results:**

Thirty-one providers participated, with 30(97%) offering both NHS and private services and 1(3%) private only. Twenty-eight providers (90%) employed a DCP, primarily a hygienist (*n* = 23,82%). While all providers referred patients to DCPs, 18 (64%) referred only for private care. Three (11%) provided NHS direct access, compared to 18 (64%) for private, citing financial and workload barriers. Remuneration methods varied, with percentage split (*n* = 16,57%) being most common. Over half of the respondents (*n* = 18,58%) felt NHS use of DCPs was unfeasible, and 21(68%) doubted that NHS contract reforms would improve DCP employment opportunities.

**Conclusion:**

Current NHS contracts may not sufficiently support DCP involvement in NHS periodontal care, indicating the need for contract reform to facilitate their broader use.

## Introduction

Periodontal diseases affect around 796 million people worldwide [1]. In the UK, the Health Security Agency reported that almost half of the UK population has a degree of periodontitis that is not reversible [2, 3]. This creates a significant service burden, which is largely supported in a primary care setting. Dental hygienists and therapists (DCPs), play essential roles in periodontal prevention and treatment provision. This approach aims to utilise varied skills, within their scope of practice, to achieve efficient care delivery in line with the utilisation of a skill mix approach recommended by the National Health System (NHS) guidance published in 2023 [4, 5].

Adoption of this model in the NHS faces regulatory, economic, and practical barriers, particularly for non-surgical periodontal therapy (NSPT) [6]. Other challenges to skill mix approach in the UK such as workforce provision, financial and patient acceptance have been previously highlighted in the literature [7]. Measures to address some of these challenges have recently resulted in availability of direct access to DCPs which means they can give patients the option to see them without having to first see a dentist and without a prescription from a dentist [5, 8, 9].

This study investigates DCPs utilization under NHS and private care models, examining barriers and opportunities for greater DCP integration. This research aimed to identify economic and structural barriers impacting DCP roles in periodontal care within NHS and private care models employed by dental providers in the South West of England, which comprises of the seven regional teams covering the counties of Bath, Wiltshire, West Bristol, Somerset, Gloucestershire, Cornwall and Isles of Scilly, Devon and Dorset [10].

## Materials and Methods

A 16-question cross-sectional online survey with no identifiable information was conducted to assess DCPs’ employment, referral types, remuneration models, and perspectives on NHS service feasibility from dental primary care providers in the South West of England. Ethical approval was granted from the University of Bristol Health Science Student Research Ethics committee (reference number 15281) on 03/07/2023. Completing the questionnaire implied consent to take part in the survey and this was highlighted in the Participant Information Sheet which was part of questionnaire introduction. The survey introduction and survey questions can be found in this paper’s supplementary information.

The survey was advertised to all dental primary care providers via South West Local Area Commissioning Team Bulletin using Microsoft Forms in August 2023 and responses collected up to the end of November 2023. The Bulletin is sent to all providers that hold an NHS contract and published on a monthly basis. The survey included questions on the nature of the practice and if it provided NHS and/or private services, as well as the size of the practice. The survey also consisted of short answer questions around the respondent’s current engagement of DCPs and remuneration models to gain an understanding of the economic models in play.

The data from the survey results were exported into a Microsoft Excel spreadsheet. Data were analysed descriptively and short answer responses gathered into tables to understand providers’ individual perspectives on DCPs integration. All answers to open questions were presented and answers to further comments on engagement of DCPs were grouped into themes after discussion among authors.

## Results

The results showed all the responses received from the 31 primary care providers highlighting their practice types, structure of DCPs’ engagement, referral patterns and dental provider perceptions. Responses are summarised in Tables 1, 2 and 3.

**Table 1:** South West primary dental care provider opinions on DCPs’ direct access.

Do DCPs provide direct access?	Reasons given
No, **private** direct access not offered.	*‘They’re not happy to see a patient without them having a full examination first’*.
	*‘They don’t want the responsibility’*.
	*‘only for hygiene, as for filling will need prescription for LA for each patient’*.
	*‘Too busy’*
	*‘Our clinicians prefer to refer patients across’*
	*‘Too busy’*
	*‘We are too busy’*
	*‘PGDs are difficult to get right for local anaesthetic especially. Hygienist confidence’*
No, **NHS** direct access not offered	*‘It’s not viable’*
	*‘They don’t want the responsibility’*
	*‘It’s not financially viable’*
	*‘No available UDAs’*
	*‘We have a limited NHS contract and can only see non fee paying patients’*
	*‘as for need for prescription from dentist when needs LA for filling, referral system is complicated’*
	*‘We do not supply NHS hyg as hygienists are self employed thus charge privately’*
	*‘Not contracted on an NHS basis’*
	*‘Not cost effective, would actually run at a loss’*
	*‘Only provide Private hygiene’*
	*‘Hygienists private only’*
	*‘Child only contract’*
	*‘Hygienists appointments are all private’*
	*‘We only offere pvt hyg’*
	*‘Hygienist prefers to work privately’*
	*‘works privately only’*
	*‘Too busy & not cost effective’*
	*‘Private practice only’*
	*‘no capacity’*
	*‘We are too busy’*
	*‘as above plus financially the NHS contract in our practice couldn’t support it’*
	*‘Child only NHS contract’*
	*‘its not financially viable to have NHS patients see our hygienists’*
	*‘We have no NHS contract’*

**Table 2:** South West primary dental care provider opinions and comments on DCPs engagement on the NHS if remuneration increased.

Would a change in remuneration contracts for DCPs within the NHS allow you to employ more DCPs?	Opinion
Yes	*‘I can’t see the NHS putting in money required, so not going to happen’*
	*‘More realistic funding’*
	*‘Recruitment of DCPs are very difficult. If the workforce is increased, it would become easier and financially viable to employ more suitable candidates’*
	*‘see above’*
	*‘Would allow payment’*
	*‘If a hygiene appointment in the NHS was worth more than 1-3 UDAs this would encourage hygienists to work under the NHS contract as they would be earning nearer to what they can charge privately’*
	*‘More than 1–3 UDA’s per treatment would help as currently they can earn more working privately’*
	*‘However I doubt I would be able to recruit one!’*
	*‘To allow more options for patients’*
No	*‘I’m not sure if as do not recruit the DCPs or dentists’*

**Table 3:** Further comments on engagement of DCPs.

Themes	Comments
Financial Viability and NHS Contract Challenges	*‘The current NHS contract doesn’t allow for the use of DCPs in a financially viable way. Without contract replacement (not tweaking details), and a lot more money, NHS dentistry will die. NHSE has no funds for dentistry and no desire to change things. The government is similarly inept’.*
	*‘We refer some patients to DCPs on NHS but it does not pay. Mostly private patients. Hygienists are expensive to employ.’*
	*‘I feel reluctance to refer NHS procedures to a DCP on an NHS basis because, as an associate, this is “giving up” your “easy UDAs” - for example, I would get 3 UDAs for a simple extraction of a primary tooth that might take me 10 mins which a DCP could also do but I would also only get 3 UDAs for a challenging extraction of a grossly carious permanent molar which might take me 45 min which a DCP could not do. So, in referring all your “easy UDAs” (I.e. treatment that falls within a DCP’s scope of practice) yes you would free up space in your book but would lose the “easy UDAs” that form the upshot of the swings-and-roundabouts nature of the current NHS contract.’*
	*‘If NHS was transferred to DCPs that would be balanced by a cut in funding to Associates as the “pot” is finite.’*
	*‘We operate a model of shared care as far as possible with dental therapists and dentist providing examinations along with hygiene in a structured 30 min appointment. They have been well accepted using my personal recommendation to book with therapist for recall examinations. NHS remuneration is challenging as fees are so low.’*
	*‘I am unsure in practices with low UDA values how the use of DCPs can work whilst meeting their wage expectations. Also a lack of access to the NHS pension is an issue’*
Renumeration and scope of practice limitations	*‘adjusting pay for NHS DCPs, ability to prescribe, engagement from dentist to establish feasible referral network’*
Workforce and recruitment challenges	*‘Projects to increase training positions using experienced dental nurses and the apprenticeship model would be a positive step forward in addressing the shortage, the relative high wages and the availability of suitable candidates’*
	*‘Due to a lack of available hours I cannot take any more DCP’s at present’*
	*‘I have advertised for hygienist and therapist but with no luck. Recruitment is difficult in rural areas.’*
	*‘We have been trying to recruit for more hygienist availability for a long time but have been unsuccessful. Candidates are either unsuitable as they are overseas dentists wanting to work as hygienists whilst waiting to sit the IQA or more interested in facial aesthetics than dental hygiene. More Hygienists and Therapists working in general practice would be great but I think this could only work in a large multi clinic setup and on a salaried basis. Fee per item gives the wrong incentive and the UDA system is not fit for purpose and not likely to be meaningfully reformed. For this type of skill mix to work with NHS provision, the surgeries will need to be nationalised and brought into the mainstream NHS and staff salaried. The surgery costs are similar to a dentists as you need the same basic equipment, a Nurse, support team, compliance cost and so on’*
	*‘Not suited in our practice to use DCP for more than hygiene purposes’*
Advantages of DCP engagement	*‘I think a change would not only encourage hygienists to work on the NHS but also help patients, as we always have patients asking if they can see a hygienist on the NHS. Especially those on low incomes and benefits.’*
	*‘Encouragement for hygienists to work on the NHS would also help patients on exemptions to access hygienist services.’*
Miscellaneous	*‘nearing retirement, leave it to someone else!’*

### Practice types and DCP engagement

- Practice mix: thirty respondents (96.77%) offered both NHS and private services, with only 1 practice (3.23%) operating as private-only. None of the respondents provided NHS-only services.
- Practice size: number of chairs in participants’ practices varied, with 8 (25.81%) having two chairs, 4 (12.90%) having three chairs, 9 (29.03%) having four chairs, 3 (9.68%) having five chairs, 4 (12.90%) having six chairs and 3 (9.68%) having seven chairs.
- DCP utilization: twenty-eight providers (90.32%) engaged DCPs, while 3 (9.68%) did not engage DCPs in their practice.

Out of those that engaged DCPs, 23 (82.14%) engaged a hygienist only, 4 (14.29%) engaged a mix of hygienists and therapists while 1 provider did not specify whether they engaged hygienists only or a mix of hygienists and therapists.

Those providers (*n* = 3) that did not engage DCPs highlighted recruitment issues, (*n* = 1), surgery capacity (*n* = 1) and the practice being a *“sedation practice”* (*n* = 1) as limitations to engaging DCPs.

### Referral patterns and treatment access

- Referral type
  In providers that engaged a DCPs (*n* = 28), clinicians referred treatment to the DCPs. Referrals to DCPs were predominantly private (*n* = 18, 64.29%), followed by mixed NHS/private referrals (*n* = 10, 35.71%). None of providers referrals were NHS only.
- Direct Access
  Private direct access by DCPs was provided in 21 of the participating providers (75.00%) that engaged DCPs. While NHS direct access was only provided in 3 (10.71%) of the participating providers that engaged a DCP.
  Reasons for direct access challenges either privately or on the NHS are highlighted on Table 1.

### Remuneration models

- Compensation structures: Sixteen providers (57.14%) responded DCPs were remunerated through percentage splits and 9 providers (32.14%) remunerated their DCPs through hourly rates. Three providers (10.71%) remunerated DCPs using other models: a hybrid model of hourly rate and percentage split, dependent on the clinician (*n* = 1) or type of treatment (*n* = 1) and salaried positions (*n* = 1).

### NHS feasibility

- Perceived feasibility: Eighteen providers (58.06%) viewed DCPs’ utilization on the NHS as unfeasible.
- Change in NHS renumeration contract views: Twenty-one providers (67.74%) did not think that a change in NHS renumeration contract would allow them to employ more DCPs. Further comments on renumeration contracts within the NHS are shown in Table 2.

Fifteen providers answered a final open-ended question on further comments regarding DCPs’ engagement. These were grouped into themes and highlighted in Table 3.

## Discussion

Since the 2002 introduction of dual-qualified dental hygienists and therapists and ever-increasing awareness of the importance of skill mixed practice, DCPs have played an important role in the provision of dental and periodontal primary care [4, 11]. The 2013 introduction of direct access policies for DCPs aimed to further improve patient access and streamline care by allowing DCPs to undertake procedures without the presence of a dentist, while still requiring a dentist prescription and/or prior assessment. These revisions were aimed to also benefit patients by providing clear guidance on what treatment different dental professionals were able to provide in the absence of a dentist [8]. Despite widespread awareness on DCPs scope of practice (SoP) there is limited published evidence highlighting how DCPs are being utilised within the UK health service, with a focus on treatment of periodontal disease [9]. A 2025 mixed methods study which included Bristish Society of Dental Hygiene and Therapy members as participants highlighted that both dental hygienists and dental therapists are frequently underutilised across both NHS and mixed dental practice settings. This 2025 research also highlights the need for further research to study the barriers involved [12]. These findings resonate with a 2009 survey which included General Dental Council (GDC) registered therapists which highlighted dental therapists’ concerns about being underutilised and deskilling as a result [13]. Although these publications highlight the missed opportunities of utilisation of DCPs to their full scope of practice the studies do not focus on barriers to periodontal treatment provision.

To our knowledge this is the first research to assess and identify providers’ perceived economic and structural barriers impacting DCPs’ roles in periodontal care in the South West of England. The findings of this research highlight significant economic and structural constraints on NHS DCPs utilization. Respondents indicated that private models provided greater financial viability, allowing flexibility in service fees and remuneration. NHS contract restrictions, including fixed remuneration, reliance on dentist referrals, and regulatory barriers for certain treatments, were noted as primary limitations to DCPs engagement in NHS periodontal care. None of the respondents listed lack of knowledge on the scope of practice as a barrier to the provision of periodontal treatment by DCPs, which contrasts with the findings of the 2020 review of the scope of practice. The 2020 review highlighted that dental professionals were confident and clear on their own scope but lacked confidence when it came to the scope of other dental professionals. In this report, it was also highlighted that DCPs were more familiar with the SoP guidance document than dentists [9]. In our survey, some of the respondents highlighted that some DCPs do not feel confident to see patients without a referral and that in the respondents’ views DCPs did not want the responsibility that comes with direct access. Since our survey was anonymous and did not ask respondents to provide information on their current role (dentist, principal, dental hygienist, dental therapist etc.) it is difficult to conclude if these perceived barriers were in line with the SoP guidance findings or not [9].

Recurring themes in the responses highlighted that despite legislative changes encouraging DCPs involvement, NHS remuneration models at the time of questionnaire completion still did not compete with the financial viability encountered in private practice, where providers can set treatment fees based on services delivered. Providers in this study further highlighted that NHS remuneration structures, including fixed bands for services, create challenges in sharing compensation between dentists and DCPs, particularly when multiple providers contribute to the same treatment course. Such remuneration limits deter NHS providers from engaging DCPs. This survey focused on hygienists and therapists; however, future studies could look at involvement of dental nurses according to their scope of practice and appropriate training to support periodontal treatment by delivering “oral health education and oral health promotion” and “measuring and recording plaque indices” under direction of another registrant [5, 9].

Research in the US has previously shown that reducing financial barriers improved periodontal care compliance and provision [14]. Therefore, more streamlined periodontal care provision through the NHS is likely to improve access to the ever-increasing population treatment needs in line with the most recent periodontal treatment guidelines, where prevention and risk factor control play a key role in overall periodontal and general health treatment and outcomes [15–19].

All answers to open questions were presented in this manuscript. Comments on engagement of DCPs were grouped into themes after discussion among authors, but some other themes could be suggested. Some of the respondents listed difficulty in recruitment of DCPs as a barrier to the provision of periodontal treatment through DCPs. Despite several reports highlighting recruitment and retention challenges in the NHS dentistry and across the whole dental team, there is no current published data that focuses on challenges related to DCPs recruitment in dentistry (NHS and private) [20, 21].

The main limitations to this study include the sample size and lack of breadth in geographical area, since the responses were collected from a total of 31 providers in one region in the UK. This limits the extrapolation of the findings to the wider South West and more so to UK dental periodontal treatment providers, since challenges and opportunities experienced in different regions or service provision niches can vary [22]. The survey was sent to all the South West providers, but respondents are likely those with stronger views on the subject. This study focused on use of dental hygienists and dental therapists as a joint periodontal care provider group and findings are limited to this combined group. As previously highlighted, other possible periodontal care providers, such as dental nurses, have not been explored in this survey. Expanding the survey to cover perspective on utilisation of separate dental care professionals individually and adoption of other study designs, such as a formal qualitative study, would highlight different perspectives of challenges or opportunities not explored in this study. Despite these limitations and the inability to determine causation through the chosen study design, this study provides important information in an infrequently published topic. Knowledge gained in the insights of dental care providers is important in shaping future implementable changes in dental periodontal care provision, especially in the current NHS-crises climate [23].

The questionnaire was carried out prior to the 2024 legislative changes, which aim to provide more DCPs autonomy [24]. Future research in this area would highlight whether the 2024 changes improve periodontal provision, although lack of data on current provision distribution and limitations in the UK will make such comparisons difficult, further stressing the importance of this work despite its size and regional limitations. It would be of interest to study whether reduction of economic and regulatory constraints alone lead to improved access to periodontal care or whether additional barriers, which were touched upon by a few respondents on perceived as opposed to actual DCPs confidence in providing periodontal care autonomously also play a significant role in implementing a skill mix approach.

## Conclusion

This study underscores the respondents’ preference for employing DCPs in private periodontal care, where flexible economic models provide more sustainable engagement. NHS primary care settings, constrained by fixed remuneration and limited autonomy for DCPs, were highlighted as challenges towards creating a supportive environment for broader DCPs’ roles in periodontal care. Findings suggest that NHS contract reforms, focusing on flexible remuneration and reduced regulatory barriers (such as the inability to prescribe medications or local anaesthetic at the time of survey completion), could enhance DCPs utilization and support a broader skill mix in dental care. It would be of interest to assess if recent changes lead to further uptake of periodontal care by DCPs through the NHS [24].

## Supplementary information

Survey questionnaire

## Data Availability

Data will be available upon request.
