# Supplementary material for: A survey on providers’ views on utilization of dental hygienists and dental therapists delivering periodontal care in the South West of England
Source: BDJ Open. 2025 Dec 16;11:97. doi: 10.1038/s41405-025-00372-2 (PMC12708769; doi:10.1038/s41405-025-00372-2)
Supplement: Supplementary file 1 — Survey questionnaire [file 41405_2025_372_MOESM1_ESM.docx]

**Supplementary information**

**Study Survey Introduction and Questions**

This survey is aimed at the dental practice owners/providers of both NHS, private and mixed dental practices within the South West region and invites them to take part in a Masters dissertation project for the University of Bristol. It is designed to gain an insight into current provision of periodontal treatment by Dental Care Professionals (DCPs), specifically dental therapists and/or hygienists. It consists of 16 questions (a range of both multiple choice and free text answers) and will take no more than 10 minutes to complete. It would be appreciated if this survey could be completed even in the event that you do not engage DCPs as this data is also relevant and useful.

**Participant Information Sheets (PIS):**

**What will happen to my data?**

Your involvement in the study will remain confidential and is voluntary. This information will only be available to research staff and national bodies which monitor whether research studies are conducted properly. Your study data will be anonymised and not identifiable. This means that it will be given an identification number and any identifying information about you will be removed. Therefore, it will not be possible to identify you or your practice by name from any aspect of documentation or reporting for this research study. At the end of the study your data will be made “Open Data”. This means that it will be stored in an online database so that it is publicly available. Any data submitted will be anonymous and unidentifiable and you will not need to include any personal data. Any forms submitted will be done so with implied consent and you will not be able to withdraw the form once it has been submitted.

**What is open Data?**

Open data means that data are made available, free of charge, to anyone interested in the research, or who wishes to conduct their own analysis of the data. We will therefore have no control over how these data are used. However, all data will be anonymised before it is made available and therefore there will be no way to identify you or your practice from the research data.

**Why open data?**

Open access to research findings and access to data is considered best research practice and is a requirement of many funding bodies and journals. As a large proportion of research is publicly funded, the outcomes of the research should be made publicly available. Sharing data helps to maximise the impact of investment through wider use, and encourages new avenues of research.

**What are the benefits and/or disadvantages of participating in this survey?** This project hopes to gain better understanding of the utilisation of DCPs in providing supportive periodontology and how this can be improved to work successfully in both private and NHS models. Your answers are valuable to provide the current deployment methods of DCPs as well as the reasoning behind this to aid in identifying possible improvements needed. There are no risks to undertaking the survey as all answers are anonymous and unidentifiable.

**The Project**

This project is undertaken as part of the final Masters dissertation for the Health Sciences Faculty of the University of Bristol BUOLD course. This project is supervised by Dr Ana Gamboa and Dr Chris Bell. The dissertation will be available upon request. Further Information If you have any further questions or would like more information, please contact Janita Dhariwal via email at janita.dhariwal@nhs.net.

Ethics Approval Reference: 15281

**Thank you for your time.**

**Survey**

1. What is your practice type? (Please ✓ one option below)
   - Private only
   - Mixed (NHS & Private)
   - NHS only
2. How many dental chairs do you have in your practice? (Please enter number below)

______ chairs.

1. Do you currently engage DCPs (dental therapists/hygienists) in your practice? (Please ✓ one option below)
   - Yes (please continue by answering the next question)
   - No (please go to question 12)
2. If you do engage DCPs, who do you engage? (Please ✓ one option below)
   - Hygienists only
   - Therapists/Hygienists
3. Do your clinicians refer to your DCPs? (Please ✓ one option below)
   - Yes (please continue by answering the next question)
   - No (please go to question 7)
4. If Yes, are these referrals for private treatment, NHS treatment or both? (please ✓ one option below)
   - Private treatment only
   - NHS treatment only
   - Mix of NHS and private treatment
5. Do your DCPs provide direct access privately? (please ✓ one option below)
   - Yes (please go to question 9)
   - No (please continue by answering the next question)
6. If No, please can you write reasons as to why not in the box provided below.

|  |
| --- |

1. Do your DCPs provide direct access on the NHS? (Please ✓ one option below)
   - Yes (please go to question 11)
   - No (please continue by answering the next question)
2. If No, please can you write reasons as to why not in the box provided below.

|  |
| --- |

1. How are your DCPs remunerated? (please ✓ one option below)
   - Hourly rate
   - Percentage split
   - Other: please write details in the box provided below (no confidential details need to be given, just a vague description)

|  |
| --- |

1. If you do not engage a DCP, are there any specific reasons why? (Please ✓ one or more options below)
   - N/A (I do engage DCPs)
   - Surgery capacity
   - Recruitment issues
   - I am not sure what treatments a DCP can carry out under their scope of practice
   - Lack of patients
   - Other (please provide details)

|  |
| --- |

1. Do you feel it is feasible to use DCPs on the NHS? (Please ✓ one option below)
   - Yes
   - No
2. Would a change in renumeration contract for DCPs within the NHS allow you to employ (more) DCPs? (Please ✓ one option below)
   - Yes
   - No
3. If Yes, please write more details in the box provided below.

|  |
| --- |

1. If you have any further comments on engaging DCPs either privately or on the NHS, please write them on the box provided below.

|  |
| --- |
